# Tetrablemmidae, a spider family newly recorded from Cambodia (Arachnida, Araneae)

**DOI:** 10.3897/zookeys.777.24917

**Published:** 2018-07-30

**Authors:** Yucheng Lin, Shuqiang Li, Peter Jäger

**Affiliations:** 1 Key Laboratory of Bio-resources and Eco-environment (Ministry of Education), College of Life Sciences, Sichuan University, Chengdu, Sichuan 610064, China Sichuan University Chengdu China; 2 Institute of Zoology, Chinese Academy of Sciences, Beijing 100101, China Institute of Zoology, Chinese Academy of Sciences Beijing China; 3 Arachnology, Senckenberg Research Institute, Senckenberganlage 25, D-60325 Frankfurt am Main, Germany Senckenberg Research Institute Frankfurt am Main Germany

**Keywords:** Haplogynae, Indochina, new species, taxonomy, tetrablemmids

## Abstract

The family Tetrablemmidae O. Pickard-Cambridge, 1873 is reported from Cambodia for the first time. Two species belonging to the genus *Tetrablemma* O. Pickard-Cambridge, 1873 are documented as new to science: *Tetrablemmakepense***sp. n.** (male, female) from Kep Province and *Tetrablemmasokense***sp. n.** (male, female) from Battambang Province. Diagnoses, morphological descriptions, and comparative illustrations are provided.

## Introduction

The family Tetrablemmidae O. Pickard-Cambridge, 1873 is a less well-known spider group compared with those medium to large spiders in subtropical and tropical regions. The type genus, *Tetrablemma* O. Pickard-Cambridge, 1873 contains 27 species (WSC 2018) and is mainly distributed in Asia, such as China (Tong and Li 2008; Lin and Li 2010, 2014), Vietnam, Laos, Myanmar and Indonesia (Lehtinen 1981; Labarque and Grismado 2009; Lin et al. 2012), India, Nepal and Sri Lanka (Tikader 1976; Bourne 1980; Lehtinen 1981), and Angola in Africa (Brignoli 1974), Queensland, Victoria and Western Australia (Butler 1932; Burger 2008; Burger et al. 2010). Single species occur in some islands, such as Micronesia, Samoa, St. Helena, Seychelles, and Trinidad (Roewer 1963; Marples 1964; Benoit 1977; Brignoli 1978; Shear 1978; Lehtinen 1981).

Several specimens of armored spiders found in a cave and the leaf litter in Cambodia were examined, belonging to two species. Here they are described as *Tetrablemmakepense* sp. n. and *T.sokense* sp. n. Both species represent the first report of the family Tetrablemmidae from Cambodia.

## Materials and methods

All type specimens were acquired by manual collection and preserved in 75% denatured ethanol solution, and are deposited in the Senckenberg Research Institute, Frankfurt, Germany (**SMF**) and the Natural History Museum of the Sichuan University in Chengdu, China (**NHMSU**).

Specimens were examined and measured under an Olympus SZX7 stereomicroscope. Further details were studied under an Olympus BX43 compound microscope. Male palp and female genitalia were examined and photographed after they were dissected and detached from the spiders’ bodies. To reveal the configuration of spermathecae and the course of the spermophor in the palpal bulb, they were treated in lactic acid and embedded in Hoyer’s solution before taking photos. Photographs were taken with a Canon EOS 60D wide zoom digital camera (8.5 megapixels) mounted on an Olympus BX43 compound microscope. Images were combined using Helicon Focus 3.10 software (Khmelik et al. 2006).

All measurements in the text or figures are given in millimeters. Leg measurements are provided as following: total length (femur, patella, tibia, metatarsus, and tarsus). Abbreviations used in figures are as follows:

**CH** cheliceral horn;

**CP** central process;

**Cy** palpal cymbium;

**E** embolus;

**EF** epigynal fold;

**EP** epigynal pit;

**Et** embolic tip;

**IVP** inner vulval plate;

**PA** preanal scutum;

**Pb** palpal bulb;

**POG** postgenital scutum;

**Sp** spermophor;

**SR** seminal receptacle;

**Ti** palpal tibia;

**VD** vulval duct;

**VS** vulval stem.

References to figures in the cited papers are listed in lowercase (figure or figs); figures from this paper are noted with an initial capital (Figure or Figs).

## Taxonomy

### Family Tetrablemmidae O. Pickard-Cambridge, 1873

#### Genus Tetrablemma

Taxon classificationAnimaliaAraneaeTetrablemmidae

O. Pickard-Cambridge, 1873

##### Type species.

*Tetrablemmamedioculatum* O. Pickard-Cambridge, 1873 from Sri Lanka (see Lehtinen 1981).

##### Note.

The gender of *Tetrablemma* is neuter, therefore two species names described previously are amended (see below). The ending “-ensis” denotes a place, a locality or a country. It forms an adjective with two endings: “-ensis” for genera masculine or feminine in gender, and “-ense” for genera neuter in gender.

##### Composition.

Twenty-seven species were known before the current study: *T.alaus* Burger, Harvey & Stevens, 2010, *T.alterum* Roewer, 1963, *T.benoiti* (Brignoli, 1978), *T.brevidens* Tong & Li, 2008, *T.brignolii* Lehtinen, 1981, *T.deccanense* (Tikader, 1976), *T.extorre* Shear, 1978, *T.helenense* Benoit, 1977, *T.loebli* Bourne, 1980, *T.magister* Burger, 2008, *T.manggarai* Lehtinen, 1981, *T.marawula* Lehtinen, 1981, *T.mardionoi* Lehtinen, 1981, *T.medioculatum* O. Pickard-Cambridge, 1873, *T.medioculatumcochinense* Lehtinen, 1981, *T.medioculatumgangeticum* Lehtinen, 1981, *T.menglaense* Lin & Li, 2014 (species epithet corrected, original published as *T.menglaensis*), *T.namkhan* Lin, Li & Jäger, 2012, *T.nandan* Lin & Li, 2010, *T.okei* Butler, 1932, *T.phulchoki* Lehtinen, 1981, *T.rhinoceros* (Brignoli, 1974), *T.samoense* Marples, 1964, *T.thamin* Labarque & Grismado, 2009, *T.viduum* (Brignoli, 1974), *T.vietnamense* Lehtinen, 1981, *T.ziyaoense* Lin & Li, 2014 (species epithet corrected, original published as *T.ziyaoensis*).

##### Distribution and habitat.

Angola, Australia, Cambodia, China, St. Helena, India, Laos, Indonesia, Micronesia, Myanmar, Nepal, Queensland, Samoa, Seychelles, Sri Lanka, Trinidad, Victoria and Vietnam. These previously described species live mainly in the forest litter or in caves.

#### Tetrablemma kepense sp. n.

Taxon classificationAnimaliaAraneaeTetrablemmidae

http://zoobank.org/F59316C9-5C81-47D8-BA84-13B8BDD4381D

Figs 1 , 2 , 6

##### Type material.

**Holotype** ♂ (SMF), CAMBODIA: Kep Province, Kep, Phnom Kep, secondary forest, leaf litter, elevation 105 m, 10°28'50"N, 104°17'50"E, 4–8 July 2017, P. Jäger leg., sieving. **Paratypes** 7♂, 8♀ (5 ♂, 6♀ SMF; 2♂, 2♀ NHMSU), same data as holotype.

##### Etymology.

The specific name refers to the type locality; adjective.

##### Diagnosis.

**Figure 1.**
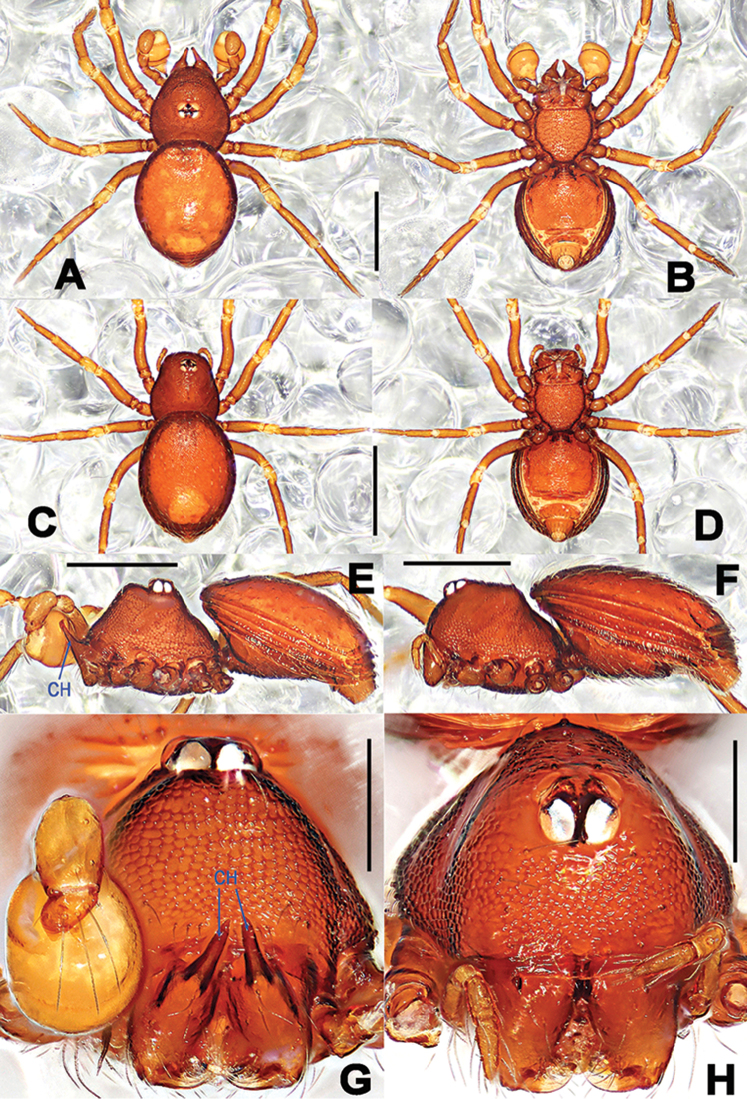
*Tetrablemmakepense* sp. n., male holotype (**A, B, E, G**) and female paratype (**C, D, F, H**). **A–F** habitus **G–H** prosoma. **A, C** dorsal **B, D** ventral **E–F** lateral **G–H** anterior. Abbreviations: CH = cheliceral horns. Scale bars: **A–F** 0.5 mm, **G–H** 0.2 mm.

**Figure 2.**
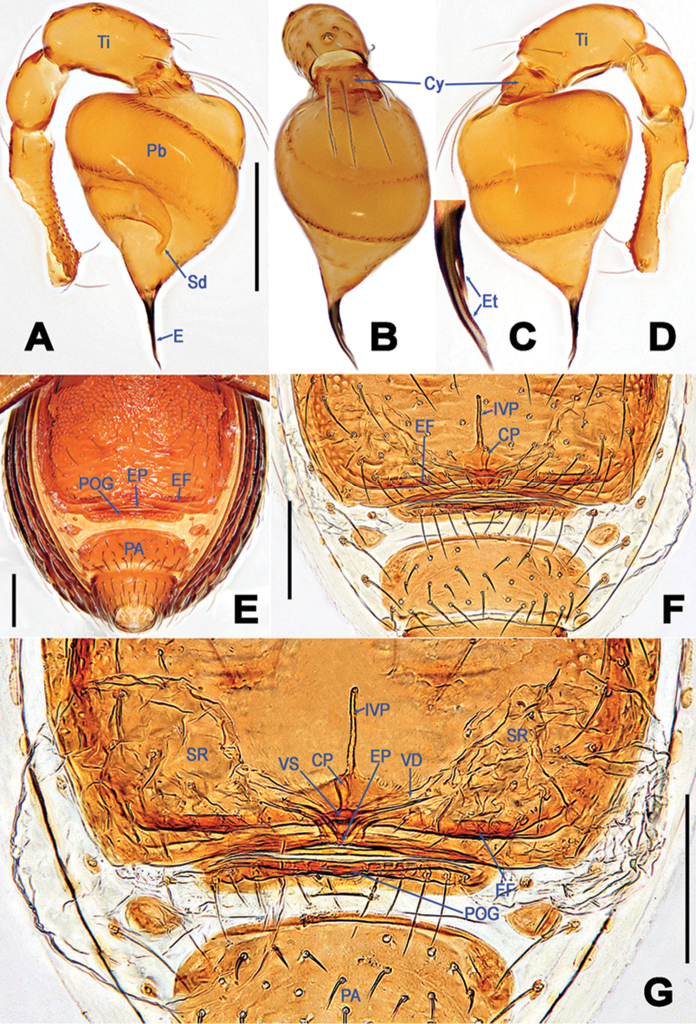
*Tetrablemmakepense* sp. n., male holotype (**A–D**) and female paratype (**E–G**). **A, B, D** left palp **C** embolic tip **E** genital area (untreated) **F–G** cleared vulva (lactic acid-treated). **A** prolateral **B, C** frontal **D** retrolateral **E, F** ventral **G** dorsal. Abbreviations: CP = central process; Cy = palpal cymbium; E = embolus; EF = epigynal fold; EP = epigynal pit; Et = embolic tip; IVP = inner vulval plate; PA = preanal scutum; Pb = palpal bulb; POG = postgenital scutum; Sd = spermophor; SR = seminal receptacle; Ti = palpal tibia; VD = vulval duct; VS = vulval stem. Scale bars: **A, B, D–G** 0.2 mm, **C** 0.05 mm.

With the exception of *T.marawula* Lehtinen, 1981, *T.brevidens* Tong & Li, 2008, *T.thamin* Labarque & Grismado, 2009, and *T.ziyaoense* Lin & Li, 2014, this new species can be distinguished from other congeners by the lack of modified teeth or tubercle on the clypeus or the ocular area in both sexes, the embolus furcated at proximally 1/3 position, the extra-long inner vulval plate nearly 3 × as long as the central process (Figs 1E–H, 2B–C, F–G). It differs from *T.marawula* by the wider bulb, the sharpened embolic tip and the longer straight inner vulval plate (Figure 2A, B, D, G vs. Lehtinen 1981: figs 266, 283); differs from *T.brevidens* by the more strongly coiled spermophor, the furcate embolus, the thinner long inner vulval plate, and shorter postgenital scutum (Figure 2A, C, G vs. Tong 2013: figure 97E–H); male differs from *T.thamin* by the narrower palpal tibia, the larger bulb, and the bifurcate embolic tip (trifurcate in *T.thamin*), female by the presence of a central process and the longer inner vulval plate (Figure 2A, C, D, G vs. Labarque and Grismado 2009: figs 1–3); and differs from *T.ziyaoense* by the converging cheliceral horns, the reticulated clypeus in both sexes (smooth in female of *T.ziyaoense*), the course and outlet position of spermophor, the precurved posterior margin of the pulmonary scutum, the thinner, straight inner vulval plate, the narrower vulval stem, and the lack of a distinct furrow on the preanal scutum (Figs 1A–H, 2A–G vs. Lin and Li 2014: figs 13A–H, 14A–D, 15A–C).

##### Description.

**Male** (holotype). Measurements: total length 1.08; carapace 0.42 long, 0.37 wide, 0.34 high; abdomen 0.70 long, 0.58 wide, 0.35 high; clypeus 0.14 high; sternum 0.28 long, 0.29 wide. Length of legs: I 1.29 (0.43, 0.12, 0.32, 0.20, 0.22); II 1.22 (0.40, 0.11, 0.30, 0.20, 0.21); III 1.08 (0.33, 0.11, 0.25, 0.19, 0.20); IV 1.38 (0.45, 0.12, 0.36, 0.23, 0.22).

Prosoma (Figure 1A, B, E, G): reddish-brown, carapace finely reticulated, marginally rugose; four eyes in one group, in posterior half of carapace, white with black ocular base, ALE>PLE, ALE and PLE adjacent; cephalic part raised; clypeus high, sloping forward, with sparse setae, marginally round; cheliceral horns long, distal tip sharp, and tilted, cheliceral lamina developed; endites basally wide, distally narrow, labium triangular, distally blunt; sternum irregularly reticulated, scutellate, marginally strongly rugose, posterior corner truncated. Legs: yellowish-orange, cuticle striated; tibiae I–III with three trichobothria, four on tibia IV, and one on metatarsi I–IV. Opisthosoma: pale reddish-brown, dorsal scutum oval, surface nearly smooth, slightly granulated, posteriorly truncated; ventral scutum reticulated; lateral scutum I long, and exceeding beyond the posterior margin of preanal scutum; postgenital scutum straight; preanal scutum approximately oblong, slightly curved.

Palp (Figure 2A–D): femoral cuticle granulated, approx. 2.5 x longer than patella; patella normal; tibia large, swollen, approx. 1.5 x wider than femur, with a trichobothrium disto-dorsally; cymbium short, triangular from lateral view, bearing long setae; bulb pear-shaped, surface smooth; embolus tubular, distinctly sclerotized, arising from the apex of bulb; embolic tip sharply pointed, lamellar bifurcation at proximally 1/3 position; spermophor coiled into two loops, abruptly twisting to narrow, and open at the embolic tip.

**Female** (one paratype). Measurements: total length 1.16; carapace 0.44 long, 0.38 wide, 0.34 high; abdomen 0.72 long, 0.61 wide, 0.37 high; clypeus 0.13 high; sternum 0.28 long, 0.30 wide. Length of legs: I 1.26 (0.42, 0.12, 0.31, 0.20, 0.21); II 1.19 (0.40, 0.11, 0.29, 0.19, 0.20); III 1.09 (0.34, 0.11, 0.26, 0.18, 0.20); IV 1.34 (0.44, 0.12, 0.35, 0.22, 0.21).

Prosoma (Figure 1C, D, F, H): coloration and modification as in male, but ocular area in anterior part of the carapace, chelicerae without long horn, only a small nodule fronto-proximally. Clypeus slightly lower than in male, palps distinctly reduced, palpal tibia with a dorsal trichobothrium. Legs as in male. Opisthosoma: dorsal and ventral scuta as in male, except for darker coloration; lateral scutum I long, extending beyond posterior margin of preanal scutum; postgenital scutum long, bearing a row of long setae, mesally narrow, laterally wide; preanal scutum more smooth, wider than long, nearly rectangular, with sparse stiff setae.

Epigyne and vulva (Figure 2F, G): epigynal fold long and obvious; epigynal pit distinct, gap-shaped, opening at the posterior margin of the pulmonary scutum; vulval stem sclerotized; vulval ducts weakly sclerotized, translucent, widely “V”-shaped, apical end connected to seminal receptacles; seminal receptacles inflated, saccular, transparent; inner vulval plate extra-long, straightly tubular, broad at base; central process short, proximally contracted, distally swollen.

##### Habitat.

Forest leaf litter.

##### Distribution.

Known only from the type locality (Figure 6).

#### Tetrablemma sokense sp. n.

Taxon classificationAnimaliaAraneaeTetrablemmidae

http://zoobank.org/34822C18-C945-4661-B08A-3528237E073D

Figs 3 , 4 , 5 , 6

##### Type material.

**Holotype** ♂ (SMF), CAMBODIA: Battambang Province, SW Battambang, Phnom Romsay Sok, an anonymous cave, elevation 100 m, 12°57'28"N, 103°6'32"E, 19 July 2017, P. Jäger & S. Münnich leg. **Paratypes** 7♂, 20♀ (4♂, 10♀; SMF 3♂, 10♀ NHMSU), same data as holotype.

##### Etymology.

The specific name refers to the type locality; adjective.

##### Diagnosis.

**Figure 3.**
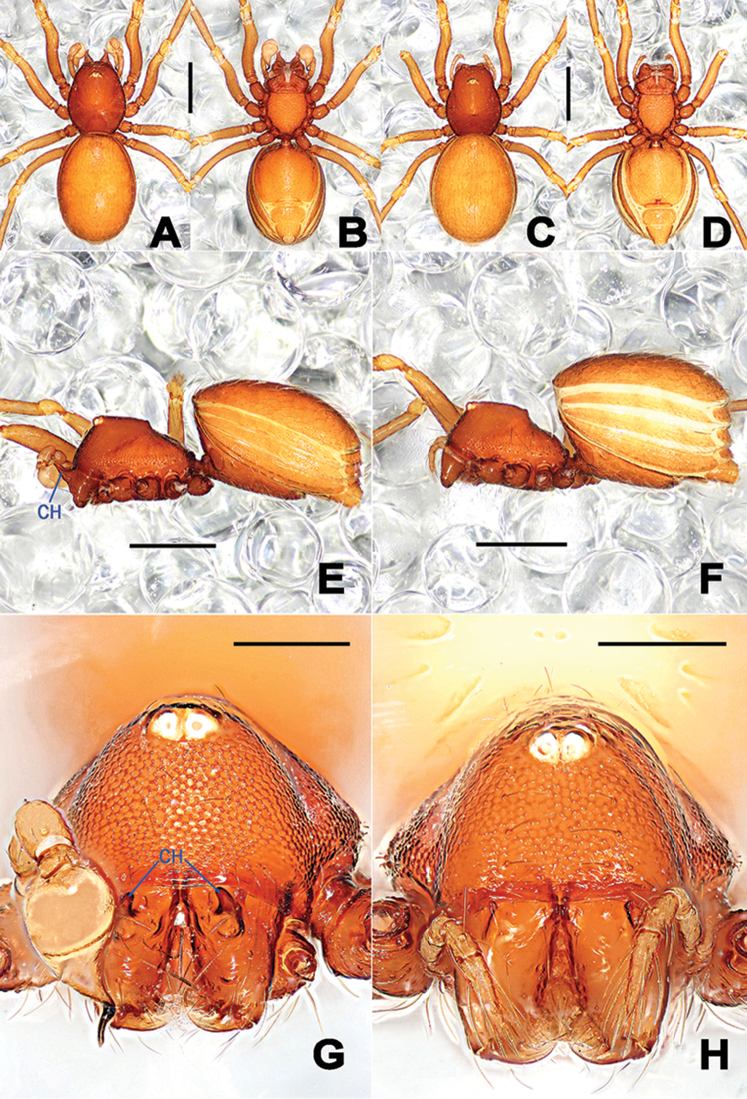
*Tetrablemmasokense* sp. n., male holotype (**A, B, E, G**) and female paratype (**C, D, F, H**). **A–F** habitus **G–H** prosoma. **A, C** dorsal **B, D** ventral **E–F** lateral **G–H** anterior. Abbreviations: CH = cheliceral horns. Scale bars: **A–F** 0.5 mm, **G, H** 0.2 mm.

**Figure 4.**
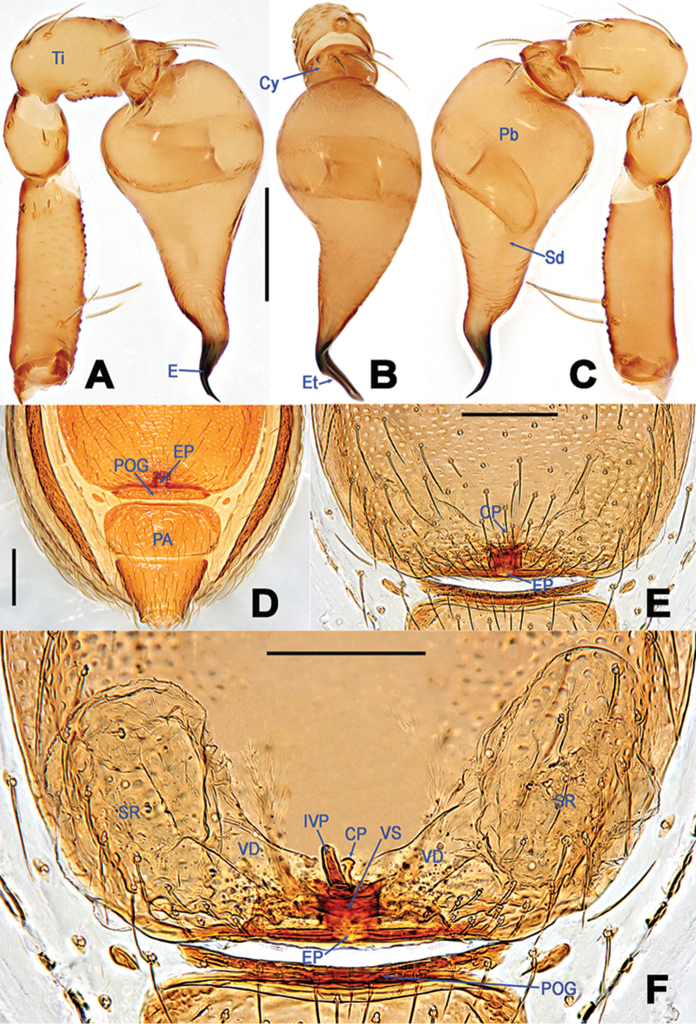
*Tetrablemmasokense* sp. n., male holotype (**A–C**) and female paratype (**D–F**). **A–C** left palp **D** genital area (untreated) **E, F** cleared vulva (lactic acid-treated). **A** prolateral **B** frontal **C** retrolateral **D–E** ventral **F** dorsal. Abbreviations: CP = central process; Cy = palpal cymbium; E = embolus; EP = epigynal pit; Et = embolic tip; IVP = inner vulval plate; PA = preanal scutum; Pb = palpal bulb; POG = postgenital scutum; Sd = spermophor; SR = seminal receptacle; Ti = palpal tibia; VD = vulval duct; VS = vulval stem. Scale bars: 0.2 mm.

This new species can be distinguished from other congeners with the exception of *T.loebli* Bourne, 1980 and *T.nandan* Lin & Li, 2010 by the protruding ocular area of carapace and the vestigial four eyespots in both sexes, the exceptionally slender bulb, and the absence of an epigynal fold in the female vulva (Figs 3A–H, 4A–F). *T.sokense* sp. n. seems similar to *T.loebli* and *T.nandan* in the appearance of habitus, the shape and configuration of genital organs, but differs from *T.loebli* by the reduced eyes without any black pigments in both sexes, the straight cheliceral horn in lateral view, the narrower bulb, the shorter, twisted embolus, the stubby inner vulval plate, and the falcate central process (Figure 3A, C, E–H vs. Bourne 1980: figs 9–10, 14, 16; Lehtinen 1981: figs 219, 221–223); and differs from *T.nandan* by the converged eyes and flat cephalic area in both sexes, the regular, not swollen palpal femur and tibia, the swollen bulb with a more distorted embolus, the shorter, fingerlike inner vulval plate and the lack of epigynal fold (Figs 3E–H, 4A–F vs. Lin and Li 2010: figs 38–39, 44–49).

##### Description.

**Male** (holotype). Measurements: total length 1.48; carapace 0.53 long, 0.50 wide, 0.48 high; abdomen 1.07 long, 0.60 wide, 0.53 high; clypeus 0.15 high; sternum 0.31 long, 0.29 wide. Length of legs: I 1.42 (0.47, 0.13, 0.35, 0.24, 0.23); II 1.35 (0.44, 0.12, 0.34, 0.23, 0.22); III 1.15 (0.36, 0.11, 0.27, 0.20, 0.21); IV 1.44 (0.49, 0.12, 0.37, 0.23, 0.23).

Prosoma (Figure 3A–B, E, G): reddish-brown, carapace finely reticulated, marginally rugose; four white eyes, as vestigial eyespots in a group, ocular area in anterior half of carapace; ALE>PLE, ALE and PLE adjacent; cephalic part raised, flat top; clypeus high, sloping forward, with sparse setae, marginally round; cheliceral horns long, straight, distal tip sharp, cheliceral lamina developed; endites basally wide, distally narrow, labium trapezoidal, distally truncated; sternum finely reticulated, marginally strongly rugose, posterior end truncated. Legs: yellowish-orange, cuticle striated; tibiae I–III with three trichobothria, two on tibia IV, and one on metatarsi I–IV. Opisthosoma: orange, dorsal scutum long oval, surface reticulated, weakly granulated, bearing sparse long setae; ventral scutum reticulated; lateral scutum I long, and exceeding beyond the posterior margin of preanal scutum; postgenital scutum straight, banded, mesally wide and laterally narrow; preanal scutum flat, nearly oval, with long setae.

Palp (Figure 4A–C): femoral cuticle ventrally granulated, approx. 2.2 × longer than patella; patella short and smooth, distinctly longer than cymbium; tibia wide, swollen, approx. 1.2 x wider than femur, with a distal-dorsally trichobothrium; cymbium short, constricted, bearing sparse long setae; bulb long pear-shaped, surface of dilated area smooth, surface of sub-distal area rugose; embolus strongly sclerotized, starting from the apex of bulb; embolic tip cuneate, twisted, sharply pointed; spermophor coiled into a loop in bulb, abruptly twisting to narrow, and open at the embolic tip.

**Female** (one paratype). Measurements: total length 1.52; carapace 0.55 long, 0.49 wide, 0.47 high; abdomen 1.03 long, 0.67 wide, 0.60 high; clypeus 0.14 high; sternum 0.32 long, 0.28 wide. Length of legs: I 1.39 (0.46, 0.12, 0.37, 0.23, 0.21); II 1.30 (0.42, 0.12, 0.33, 0.22, 0.21); III 1.18 (0.37, 0.11, 0.28, 0.22, 0.20); IV 1.45 (0.48, 0.12, 0.38, 0.24, 0.23).

**Figure 5.**
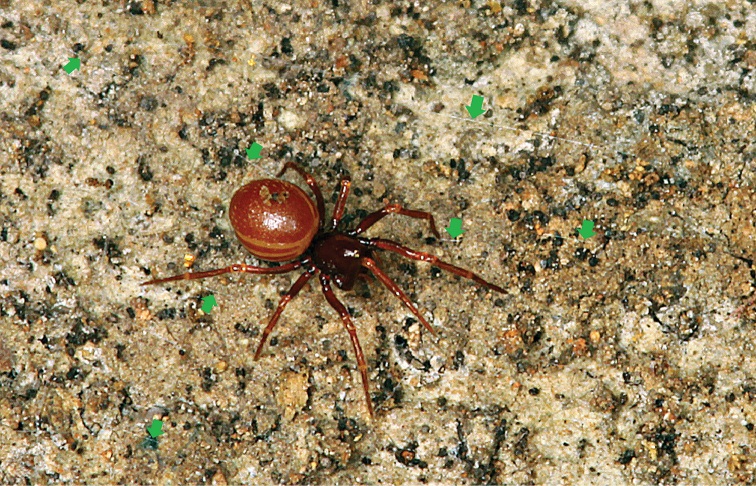
*Tetrablemmasokense* sp. n., live female in cave, Cambodia (green arrows indicating silken strands, photograph by P. Jäger).

Prosoma (Figs 3C–D, F, H, 5): coloration and modification as in male, but instead cheliceral horns a small nodule fronto-proximally. Clypeus lower than in male, palps distinctly reduced, palpal tibia with a dorsal trichobothrium. Legs as in male. Opisthosoma: dorsal and ventral scuta as in male; lateral scutum I long, extending beyond posterior margin of preanal scutum; postgenital scutum long, straight, bearing a row of long setae, mesally wide, laterally narrow; preanal scutum weakly sculptured, wider than long, nearly rectangular, with sparse stiff setae.

Epigyne and vulva (Figure 4E–F): epigynal fold absent; epigynal pit indistinct, oval, opening at the posterior margin of the pulmonary scutum; vulval stem short, columnar, strongly sclerotized; vulval ducts wide, translucent, widely “V”-shape; seminal receptacles saccular, transparent; inner vulval plate fingerlike, distinctly sclerotized, slightly tilting; central process small, weakly sclerotized, shorter than inner vulval plate, apically falcate.

##### Habitat and biology.

Cave. The cave entrance was ca. 80 altitudinal meters above the ground at an isolated limestone hill. Almost under every stone only one spider was found; silken strands used by the spiders may be the reason for this territoriality (Figure 5). It was the highest density of Tetrablemmidae observed in a cave by the last author. Besides Tetrablemmidae, there were Oonopidae (also found under stones), Pholcidae, Sparassidae, Uloboridae, Hersiliidae, Agelenidae, Amblypygi and Isopoda.

##### Distribution.

**Figure 6.**
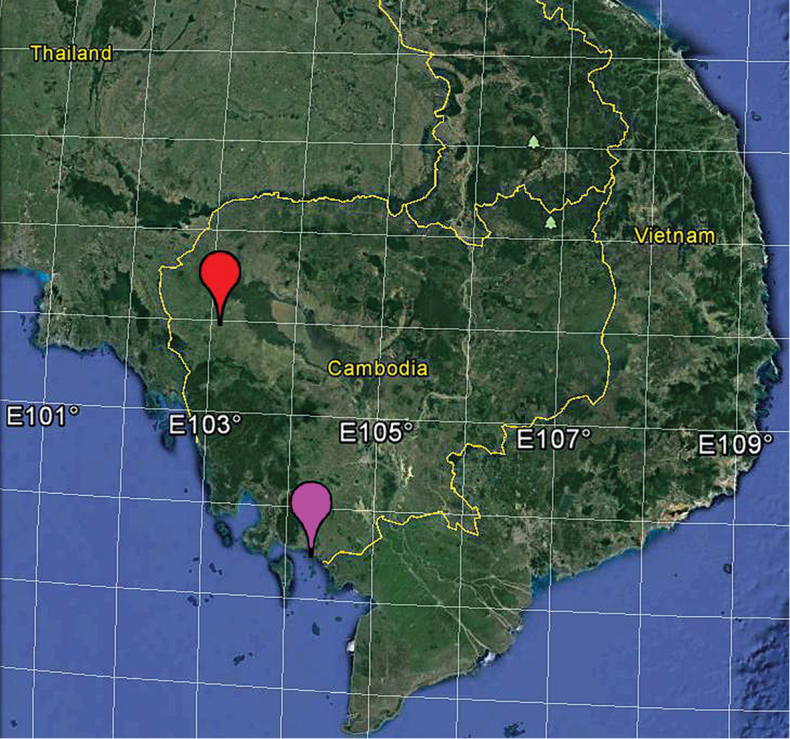
Distribution records of *Tetrablemmakepense* sp. n. (pink balloon) and *Tetrablemmasokense* sp. n. (red balloon) in Cambodia.

Known only from the type locality (Figure 6).

## Supplementary Material

XML Treatment for Tetrablemma

XML Treatment for Tetrablemma kepense

XML Treatment for Tetrablemma sokense
